# Unambiguous Ex Situ and in Cell 2D ^13^C Solid-State NMR Characterization of Starch and Its Constituents

**DOI:** 10.3390/ijms19123817

**Published:** 2018-11-30

**Authors:** Alexandre Poulhazan, Alexandre A. Arnold, Dror E. Warschawski, Isabelle Marcotte

**Affiliations:** 1 Department of Chemistry, Université du Québec à Montréal, Downtown Station, P.O. Box 8888, Montreal, QC H3C 3P8, Canada; poulhazan.alexandre@courrier.uqam.ca (A.P.); arnold.alexandre@uqam.ca (A.A.A.); Dror.Warschawski@ibpc.fr (D.E.W.); 2 Laboratoire de Biologie Physico-Chimique des Protéines Membranaires, UMR 7099, CNRS, Université Paris Diderot and IBPC, 13 rue Pierre et Marie-Curie, 75005 Paris, France

**Keywords:** whole cell NMR, magic-angle spinning, 2D INADEQUATE, crystalline and amorphous starch

## Abstract

Starch is the most abundant energy storage molecule in plants and is an essential part of the human diet. This glucose polymer is composed of amorphous and crystalline domains in different forms (A and B types) with specific physicochemical properties that determine its bioavailability for an organism, as well as its value in the food industry. Using two-dimensional (2D) high resolution solid-state nuclear magnetic resonance (SS-NMR) on ^13^C-labelled starches that were obtained from *Chlamydomonas reinhardtii* microalgae, we established a complete and unambiguous assignment for starch and its constituents (amylopectin and amylose) in the two crystalline forms and in the amorphous state. We also assigned so far unreported non-reducing end groups and assessed starch chain length, crystallinity and amylose content. Starch was then characterized in situ, i.e., by ^13^C solid-state NMR of intact microalgal cells. Our in-cell methodology also enabled the identification of the effect of nitrogen starvation on starch metabolism. This work shows how solid-state NMR can enable the identification of starch structure, chemical modifications and biosynthesis in situ in intact microorganisms, eliminating time consuming and potentially altering purification steps.

## 1. Introduction

Starch is, with cellulose, the most abundant carbohydrate that is found in nature. Composed of a polymer of glucose in semicrystalline granules, it is the major form of energy storage for plants [1]. It is also the main energy source in most animal diets and is involved in various food industry processes. Because they are linked with diseases, starches that are resistant to digestive enzymes have been the focus of a growing research emphasis [2]. In addition to its importance in nutrition, starch can be used as an environmentally-friendly low-cost material with no apparent toxicity and can be functionalized for a wide range of applications, such as adhesives, biofilms, biodegradable plastics, pharmacology, etc. Recently, for example, starch was designed for drug delivery using hydroxymethylated material [3].

**Figure 1:**
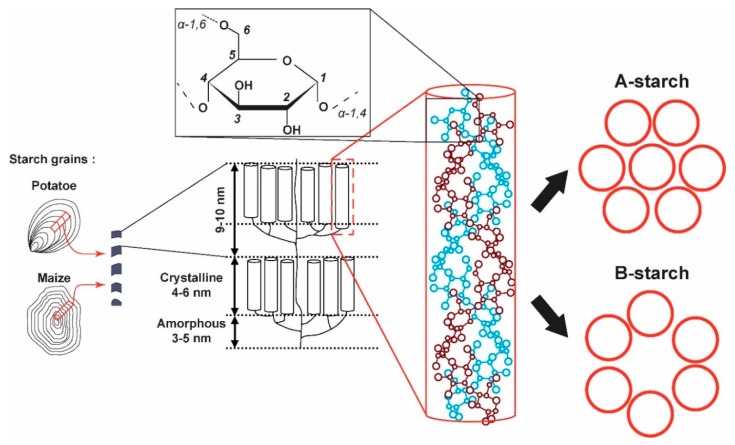
Multiscale representation of starch, from left to right: morphology of starch granules [17], crystalline and amorphous regions, double helices and packing of double helices in crystalline forms A and B.

In the Plantae, starch is stored in grains, structured on different scales, as illustrated in Figure 1. At a micrometer (µm) scale, starch granules are made of amorphous and crystalline regions. Starch is water insoluble, making it easy to purify, and consists mainly of highly branched amylopectin (70 to 85% by weight of short α-1,4 chains with numerous α-1,6-d-glucan linkages) and linear amylose (15 to 30% by weight of long α-1,4-d-glucan with few α-1,6-d-linkage) [4]. Starch is thus a semi-crystalline network of amylose and amylopectin chains stranded into a double-helical structure held by hydrogen bonds. These helices have 6 glucose residues per turn and a pitch of 2.1 nanometer (nm) and can adopt two different crystalline packings identified as the A and B-types [4]. However, many vegetables possess starch grains that contain a mixture of A and B-types categorized as the C-type [4]. These three forms were first identified by X-ray diffraction (XRD) analysis [5]. According to previous works [6,7], the main difference between A and B-type starches is the relative positions of the starch double-stranded helices. In the A-type structure, left-handed parallel-stranded double helices are closely packed into a B2 space group [8,9], while in the B-type, helices are packed into a hexagonal unit cell corresponding to a P6_1_ space group [10], forming a more hydrated structure.

One of the limitations of the starch industry is the difficulty to control the final product quality. Today, native starch and its derivatives are most frequently characterized by powder X-ray diffraction [4,11], often in combination with SS-NMR (solid-state nuclear magnetic resonance) [12,13,14,15,16]. NMR has the advantage of being a non-destructive technique, thus allowing measurements on intact hydrated samples while providing information on molecular dynamics at an atomic scale, even in the case of complex biomolecules such as starch [4]. The one-dimensional (1D) study of A and B-type starches has already revealed some differences in ^13^C chemical shifts. In particular, the multiplicity of the C_1_ carbon is used to distinguish the two starch crystalline forms [12,13,14,15,16]. The C_2_ to C_5_ resonances, however, are poorly resolved on 1D spectra, and 2D SS-NMR has not yet been used to unambiguously characterize A-type starch, amorphous- and amylopectin-rich starches. To the best of our knowledge, only pure synthetic amylose has been described by 2D ^13^C NMR and proved to be in the B-type form [14].

In this work, we used the microalga *Chlamydomonas reinhardtii*—A photosynthetic model organism with well-known metabolism [18,19,20], numerous mutants and a fully sequenced genome [21,22]—for starch production and ^13^C labelling. This microorganism produces starch of the A-type after purification [11,23]. Using amylose-rich, amylopectin-rich and inhibited starch producing strains, we established a full unambiguous high-resolution assignment for all carbons in A, B and amorphous starches—An essential step in the SS-NMR study of starches and their modifications. We assigned highly crystalline amylopectin and poorly crystalline B-type amylose as well as new signals from non-reducing end groups. Beyond its chemical shift and multiplicity, the width and shape of each resonance provided additional data that were interpreted in terms of local vs. global order, correlated disorder, chain length, degree of crystallinity or amylose/amylopectin ratio. Finally, based on these characterizations, we were able to detect the resonances of starch in situ in whole *C. reinhardtii* cells, and to identify the type of starch in the storage grains, as well as the level of crystallinity. This work demonstrates how 2D ^13^C SS-NMR methodology can prove invaluable for the functional in vivo study of starch in its native environment: the cytosol.

## 2. Results and Discussion

### 2.1. 2D ^13^C SS-NMR Ex Situ Characterization of A and B Types and Amorphous Starch

To characterize starch in situ, we first needed to establish a complete unambiguous ^13^C assignment of pure A, B and amorphous starches. The A and B forms can respectively be obtained with sufficient high crystallinity using amylopectin-rich and native retrograded starches.

The A form was prepared using starch from the *st 2-1* amylopectin-rich *C. reinhardtii* strain. This strain produces starch with the highest crystallinity—about 71% according to our SS-NMR results (see Table S1). The short chains of amylopectin are reported to form double-helices which readily crystallize in the starch granules [4]. Furthermore, XRD measurements of pure amylopectin (Figure S1A) are typical of highly crystallized A-type starch [11,24]. The B form was obtained by starch retrogradation, as described in the Material and Methods section, from wild-type *C. reinhardtii*, yielding a final crystallinity of *circa* 55% as determined by SS-NMR (Table S1). Finally, amorphous starch was prepared by freeze-drying amylose-rich starch (produced by the *C. reinhardtii sta 3-3* mutant) as described by Paris et al. [15]. The amorphous nature of this sample is confirmed by NMR (about 0% crystallinity, Table S1). Interestingly, the A-type starch remained crystalline after drying, while the crystal structure of B-type starch was destroyed after drying, according to XRD experiments (Figure S1A,B). Water is thus an essential element in the crystal structure of B-type starch, as reported elsewhere [25,26].

**Figure 2:**
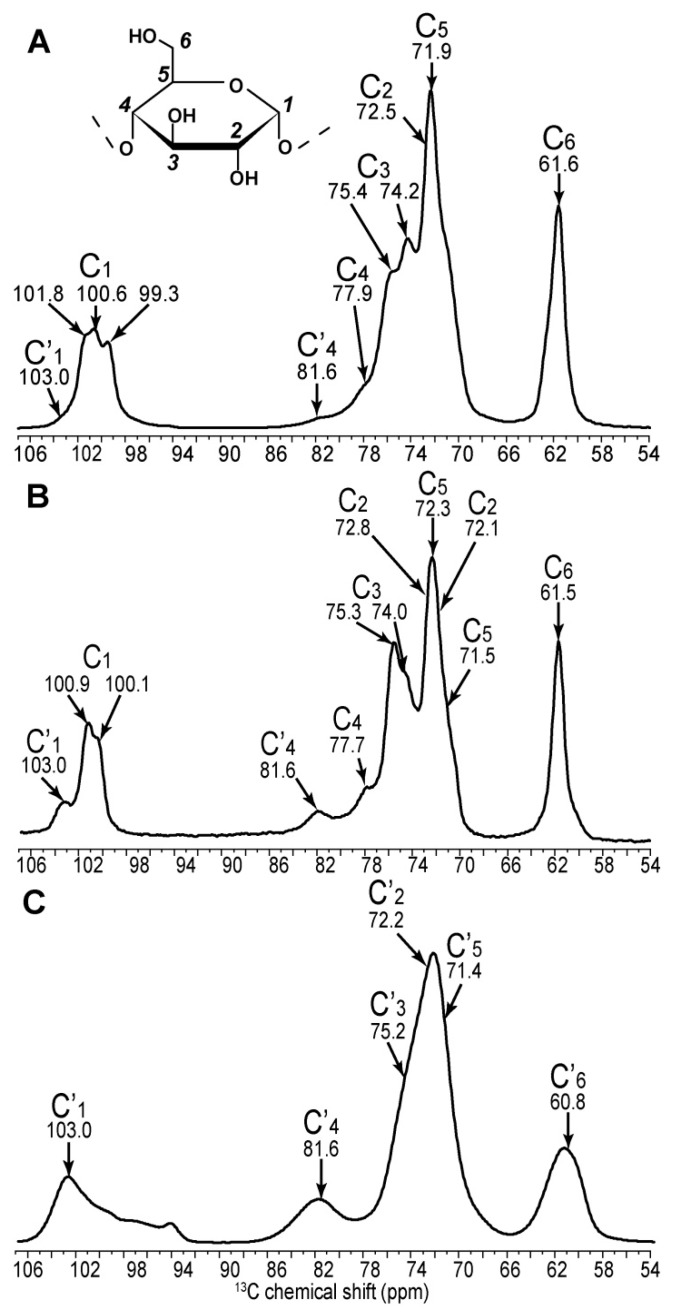
1D cross-polarisation ^13^C solid-state NMR (nuclear magnetic resonance) spectra of amylopectin (**A**), retrograded (**B**) and amorphous gelatinized (**C**) starch from *C. reinhardtii*. Assignments are extracted from 2D spectra.

As shown in Figure 2, while C_1_, C_4_ and C_6_ carbons are readily resolved on the 1D spectra of types A, B and amorphous starch, carbons 2 to 5 cannot be distinguished. Gidley and Bociek [27] demonstrated that glucose carbons 1 and 4 were more sensitive to starch conformational changes than carbons 2, 3 and 5, showing higher chemical shift dispersion under the variation of the torsion angles of the glycosidic linkage in α-(1,4) glucans. In amorphous starch, the broad distribution of conformations thus leads to a large chemical shift dispersion of the C_1_′ peak (Figure 2C). The C_1_ splitting of the crystalline forms (3 peaks for A and 2 peaks for B) have been explained as resulting from the different space groups adopted by A and B forms [27]. These spatial arrangements lead to three possible environments for carbon 1 in the A form and two for the B form.

A major improvement in resolution can be obtained using 2D methods on ^13^C labelled material, including whole cells. As we showed in a previous piece of work, microalgae can easily be fully ^13^C labelled using NaH^13^CO_3_ [28]. A higher spectral dispersion provided by the second dimension will reduce the risks of potential overlap between starch and other carbohydrate moieties in situ. The 2D INADEQUATE is an excellent experiment providing unambiguous through-bond connectivities and enhanced resolution. The robustness of this experiment and exquisite sensitivity to conformational differences has been shown in various works on disordered organic materials [29,30]. This experiment has particularly been useful in the study of cellulose [31,32]. Moreover, the double quantum (DQ) dimension provides excellent chemical shift dispersion and the experiment has, thus, also been applied to intact systems [33]. Here, we used the INADEQUATE pulse sequence in combination with proton-to-carbon polarization transfer schemes, such as Cross Polarisation (CP) or the Nuclear Overhauser effect (NOE), which is commonly exploited in solid-state NMR for signal enhancement [28,34].

**Figure 3:**
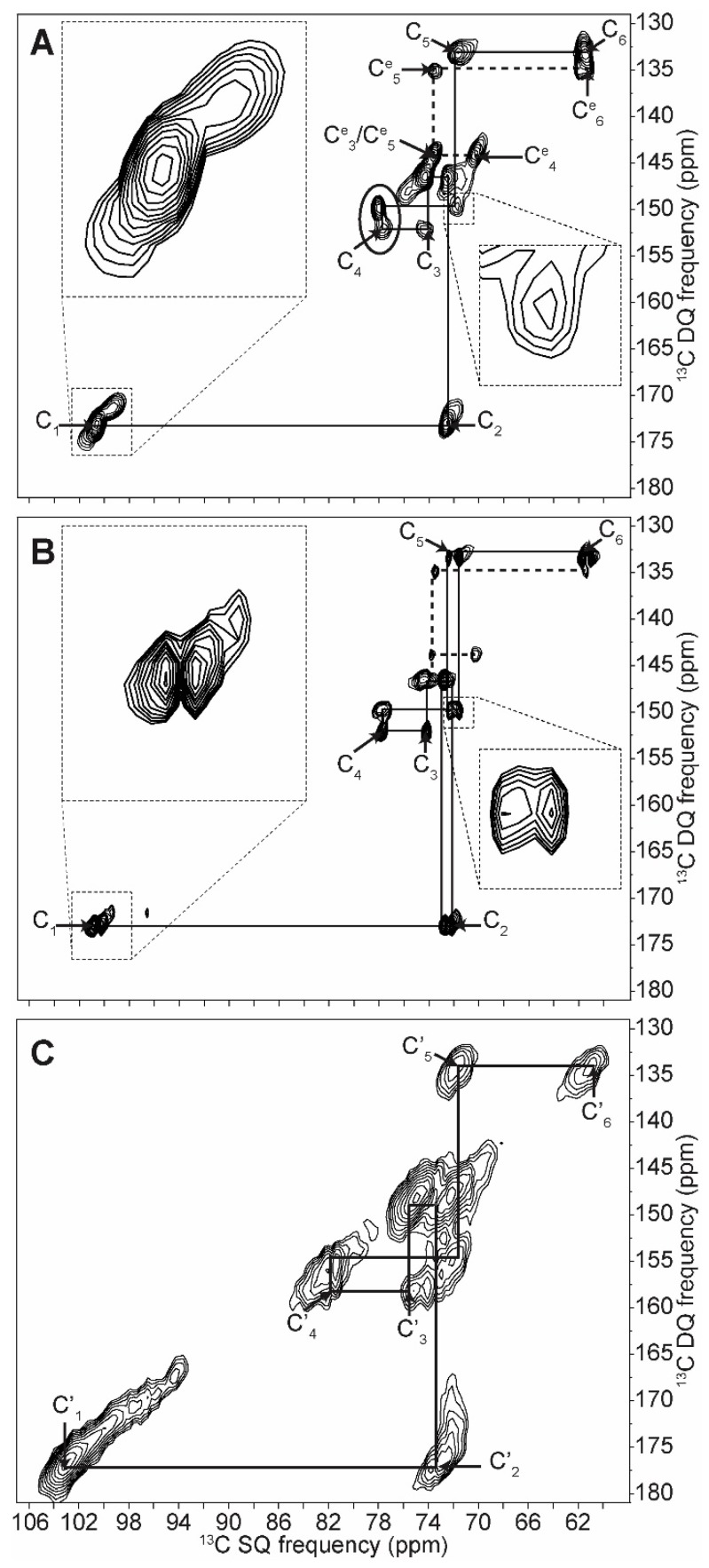
NOE (Nuclear Overhauser Effect)-INADEQUATE 2D SQ/DQ (single quantum/double quantum) ^13^C solid-state NMR spectra of pure amylopectin A-type starch (**A**), retrograded native *C. reinhardtii* B-type starch (**B**) and amorphous dry native *C. reinhardtii* starch (**C**). Continuous lines are correlation pathways for the main starch constituents (α-1,4 and α-1,6 linked glucans), while dashed lines correspond to non-reducing end groups. Zooms correspond to carbon 1 in C_1_–C_2_ correlation and to carbon 5 in the C_5_–C_4_ correlation on the left and right side zooms, respectively. On (**A**), C^e^_X_ designate carbons in non-reducing end-groups. To compare end groups signals between 2D spectra, the intensities are normalized using the C_4_ area circled in (**A**).

The 2D INADEQUATE spectra of amylopectin (A-form), retrograded *C. reinhardtii* native starch (B-form) and dry amylose (amorphous) are shown in Figure 3. The net improvement in resolution is sufficient to distinguish all carbons including carbons 2, 3 and 5, which were not unambiguously elucidated before. A complete and unambiguous assignment was thus obtained for the three starch forms, and all spin systems are reported in Table 1. The linewidths vary between ca. 100 Hz and 200 Hz for hydrated starches, thus confirming their well-ordered and dynamic nature. The linewidths of the dry amorphous starch (Figure 3C), on the other hand, can reach 500 Hz, possibly due to the dispersion of conformations and freezing out of motions in this state.

As seen on the 1D spectrum and as previously reported, the greatest chemical shift differences between starch forms are observed for the C_1_ and C_4_ carbons [27]. The C_1_ value ranges from 99.3 to 101.8 ppm in A-type starch, from 100.1 to 100.9 ppm in B-type and is equal to 103.0 ppm in amorphous starch. The difference in splitting of the C_1_ peak between A- and B-types is confirmed and extends to the C_2_ carbons for B-type starch, although this difference is lost in the following carbons, except for the C_5_ carbon of the B-form. Although chemical shift differences are small, a clear splitting is seen for C_5_ in the C_5_–C_4_ and C_6_–C_5_ correlation in the B-form spectrum, which is not present in the A-form (Figure 3). The change in C_4_ chemical shift between the two crystalline forms is small (0.2 ppm); however, a large 3.9 ppm difference is observed when starch becomes amorphous. Similarly, the difference between C_3_ chemical shifts in both crystalline forms is minor (74.2 and 74.1 ppm for A and B forms, respectively), however these can clearly be distinguished from the amorphous chemical shift (75.3 ppm). Overall, differences in chemical shifts between A and B forms are small, indicating that the torsion angles and magnetic environment are very similar. The structures are thus *locally equivalent* and the notable differences that we detected for the C_1_ resonances, and which we showed to partially extend to other carbons, result from differences on a longer scale, such as the symmetry of the crystal lattice.

Most importantly, all 2D spectra reveal a clearly distinct spin system (around 100.5, 72.5, 73.5, 70.2, 73.5, 61.3 ppm) that has never been reported in natural starches to the best of our knowledge, most likely because they are almost undetectable on 1D spectra (except for a shoulder near the C_5_ carbon). These resonances cannot arise from soluble molecules, such as short carbohydrate oligomers, because they would have been eliminated in the various washing steps of our samples. On the other hand, the chemical shifts of this system of correlated carbons are in excellent agreement with those reported for the end-groups of synthetic alpha dextrins [35] and can, therefore, most certainly be assigned to non-reducing terminal glucose groups.

**Figure 4:**
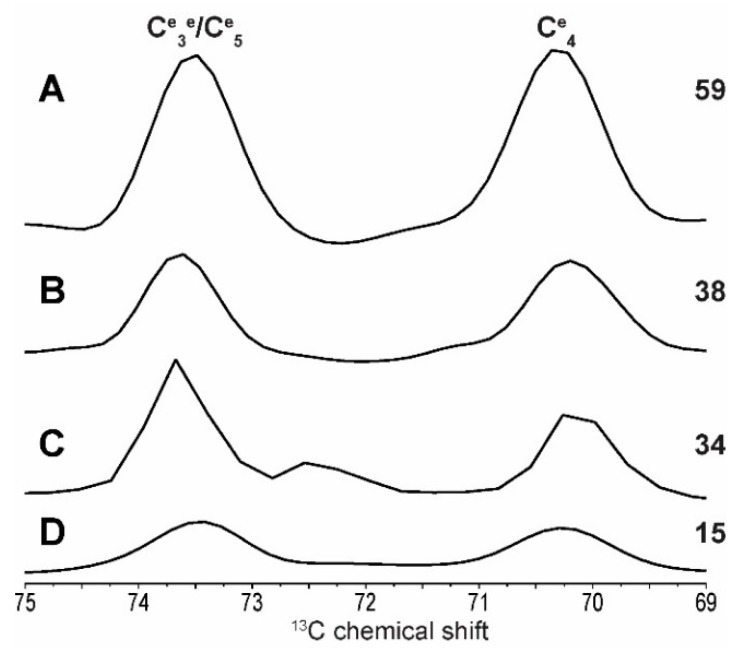
Traces corresponding to the C^e^_3_/C^e^_5_–C^e^_4_ correlation in non-reducing end groups are extracted from 2D spectra with the C_4_ area (see Figure 3) arbitrarily set to 100. Amylopectin-rich (**A**), native (**B**), native retrograded (**C**) and amylose-rich (**D**) starches traces are presented. C^e^_4_ intensities are indicated on the right.

As such, the intensity of the C^e^_4_ resonance (Figure 4) cannot allow us to directly measure the percentage of end groups, exact chain length or branching abundance in situ, however the detection of these resonances offers the possibility of obtaining relative values. Absolute quantitative measurements are possible, however they would require prior calibration of resonance intensities with standard molecules. This information can nevertheless help localize chemical reactions in starch and it can also be very useful for the food industry as short amylopectins found in highly resistant starch are valued for their healthful properties [36]. In the context of the development of new rice mutants, for example, following the intensity of the C^e^_4_ resonance could help predict some of its functional properties and commercial values [37].

**Figure 5:**
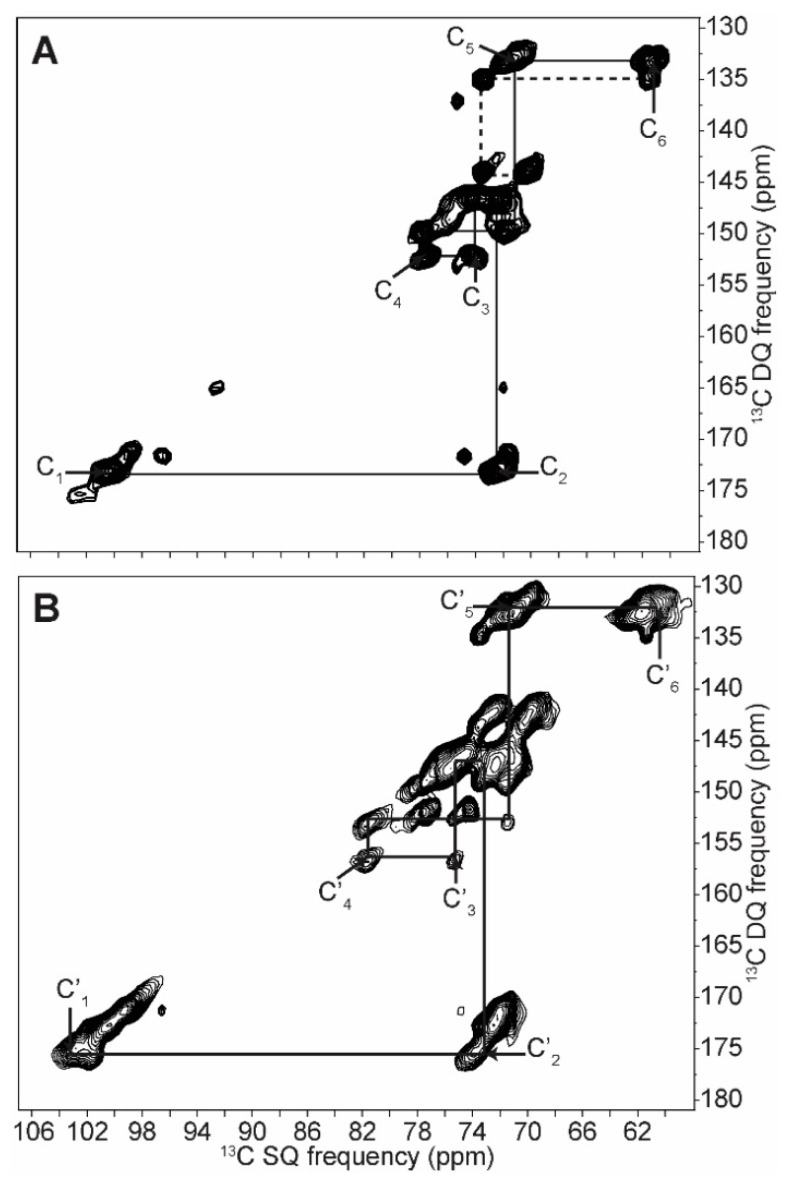
NOE (**A**) and CP (cross-polarization) (**B**) INADEQUATE ^13^C solid-state NMR spectra of native *C. reinhardtii* A-type starch. Continuous lines link correlated spins in crystalline (**A**) and amorphous (**B**) regions, while dashed lines correspond to non-reducing end groups.

As mentioned previously, we have used signal enhancement schemes, and NOE was usually favoured because it was more efficient than CP, as expected in mobile regions of biomacromolecules. One exception is hydrated amorphous starch, where CP was more efficient and CP-INADEQUATE was preferred (see Figure 5B). In hydrated amorphous starch, Paris and co-workers also used CP and explained its efficiency by the particular nature of proton-to-carbon couplings, where water polarization is transferred by spin diffusion to protons covalently bound to starch carbons in a two ^1^H reservoir model [16].

Compared to hydrated starches, the dry amorphous one showed much broader lines with an intrinsic linewidth that was at least twice that of the hydrated forms, as determined by 2D SS-NMR (see Figure S2), and as previously reported in various biological samples [38,39]. The elongated shapes of the lines in the INADEQUATE spectra, which are parallel to a 1:2 axis, reflect a distribution of conformations rather than a mere change in relaxation resulting from the reduced dynamics in the dry form. Moreover, since the resonance pattern of a given carbon is parallel to the previous and the next ones, the distribution of conformations (or disorder) is correlated from one carbon to the adjacent. In other words, the structure is purely amorphous with no regions that are more ordered than others [40]. As soon as starch is hydrated, water acts a plasticizer and chain motions average out the chemical shifts to the values given in Table 1 for hydrated amorphous starch.

To summarize this section, we characterized A and B types and amorphous starch, thus providing the most complete ^13^C NMR assignment to date, including new chemical shifts ascribed to non-reducing end groups. The widths of 1D lines, as well as the shapes of 2D resonances, are useful indications of molecular order and dynamics. Finally, the ratio of intensities between C_4_ and C^e^_4_ enables the first in situ qualitative assessment of the length of branched amylopectin or linear amylose.

### 2.2. Differentiating Between Starch Components: Amylose and Amylopectin

Macroscopically, starch has various grain shapes, however with similar architecture consisting of growth rings, blocklets and crystalline-amorphous lamellae. This glycosidic polymer is made of linear and branched sequences, respectively corresponding to amylose and amylopectin. Different ratios of amylopectin/amylose are observed in starch depending on its origin. Pure amylopectin starch has been described as highly crystalline A-type starch [4,41,42], whereas pure amylose starch has been described as having B-type and low crystallinity patterns [43]. Thus, amylose is generally considered to be in the amorphous region of starch granules [4] while amylopectin is in the crystalline region. Nevertheless, many examples show that the amylose/amylopectin ratio is not the only decisive factor determining starch type, crystallinity [44] and digestibility [2].

As expected, starch purification from amylopectin-rich strain *st 2-1* of *C. reinhardtii* leads to highly crystalline A-type starch (for XRD and NMR, see Figures S3 and S4, respectively). Indeed, the 1D ^13^C SS-NMR experiment shows that the peaks assigned to amorphous C_1_ and C_4_, respectively at 103 and 81.6 ppm, are nearly absent in amylopectin samples, thus indicating that amylose is at least partially involved in amorphous regions of starch. Furthermore, α-1,6-branched glucose was not detected here because this type of bond has an occurrence of only 5% in amylopectins [4]. As for pure amylose, which is described to poorly crystallize into the B-form [11], chemical shifts are indeed in good agreement with this starch form (Table 1). We here confirm this observation with XRD (Figure S1C) and ^13^C SS-NMR (Figures S3 and S4) on extracted starch from this strain. Moreover, the same difference could be observed in situ (data not shown). In addition, lines are sharper in the amylose spectrum than in the native and amylopectin rich A-type starch, which is in good agreement with a more amorphous/mobile amylose structure. Interestingly, amylose can also be recrystallised into A- or B-type starch [45], consistent with the higher flexibility of amylose.

This opens the way to exploring the effects of changing amylose/amylopectin ratios related to starch crystallinity and, to some extent, to its type. According to the literature, more amylose is usually more favourable to B-type starch [43], and pure amylose leads to B-type crystals in vitro [46], even if exceptions exist. Amylose/amylopectin ratio and chain length distribution are known to be critical for starch physicochemical properties, which determine their suitability for particular uses. For example, starch films properties [47,48], digestibility and starch water uptake [49] are affected by the amylose/amylopectin ratio. Thus, SS-NMR could be a rapid and efficient tool to help understand these differences.

### 2.3. In Cell Characterization of C. reinhardtii Starch

**Figure 6:**
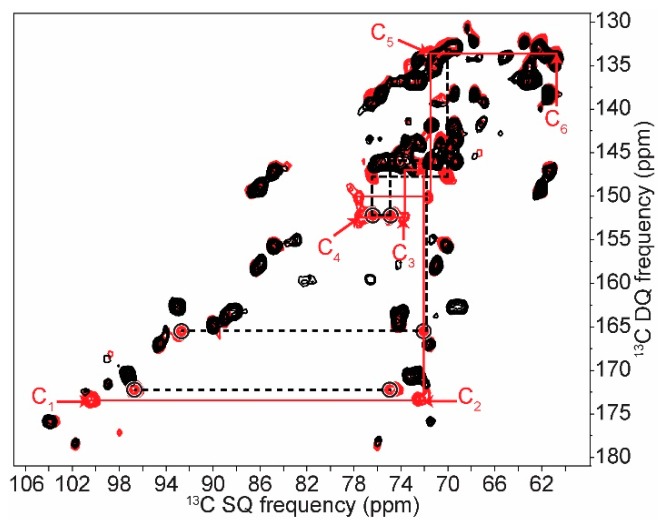
NOE-INADEQUATE of whole cell *C. reinhardtii wt* (red) and *sta 6-1* starchless (black) strains. Continuous lines indicate correlation pathways corresponding to A-type crystalline starch. Dashed lines link circled resonances from the additional correlations discussed in the text.

As will be seen in this section, our thorough characterization of extracted starch in its various forms enabled us to detect starch in cell and to identify its type and degree of crystallinity. In our previously published work, intense signals were assigned to starch in whole cells of *C. reinhardtii* [28]. Here, we further refined this assignment by exploiting the improved resolution provided by the 2D INADEQUATE experiment. As shown in Figure 6, this experiment can discriminate starch signals from those of other saccharides with the exception of carbons 6 and 3 which overlap with those of the galactolipids and/or present in the cell wall structure. A comparison of the chemical shifts obtained from the 2D ^13^C INADEQUATE and reported in Table 1 confirms that in *C. reinhardtii* cells, starch crystallizes in the A-form, which is very similar to pure amylopectin, supporting the high proportion of amylopectin in this starch (see XRD and NMR results in Figures S1A,D and S5).

Our approach can be applied to other microorganisms, however also to mutants of *C. reinhardtii*. For example, to avoid any ambiguity in our chemical shift assignments, we compared the 2D spectrum of the starchless strain *sta 6-1* to that of the wild type strain (Figure 6). The main differences confirm our assignment of starch, however we observed additional correlations in whole wild type (*wt*) cells that are absent in whole starchless mutants (see the dashed lines and circled peaks in Figure 6). These correlations can be described as one spin system of six carbons (at 92.7, 72.4, 74.9, 76.9, 70.4 and 61.4 ppm from C_1_ to C_6_) and one correlation at 96.7 ppm/75.0 ppm. We suspect them to be co-products of starch synthesis in cells that in situ SS-NMR of various *C. reinhardtii* strains has allowed to detect. An assignment of those peaks is preliminary at best, however the chemical shifts of the six correlated carbons could correspond to those observed in high-energy twisted starch helices associated with starch synthesis in the cell [50,51].

**Figure 7:**
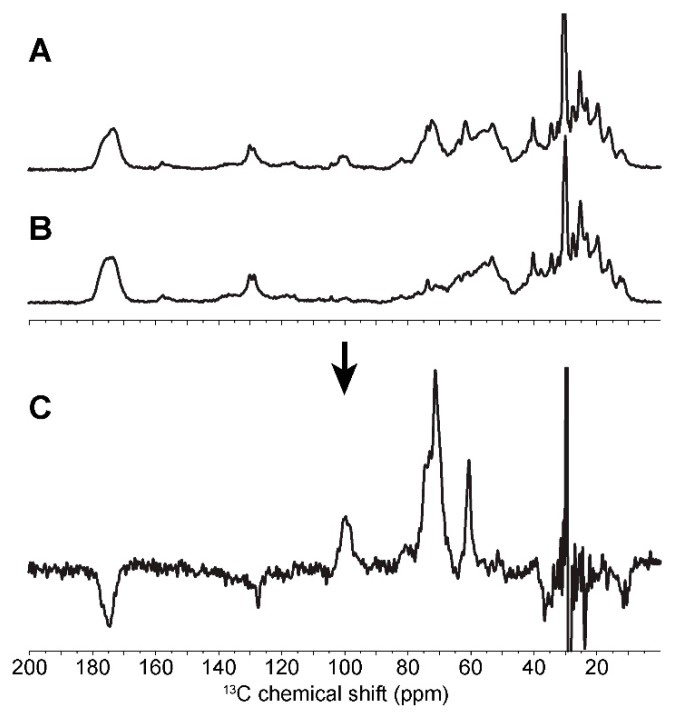
1D cross-polarisation ^13^C solid-state NMR spectra of whole *C. reinhardtii wt* cells (**A**) and *sta 6-1* starchless strain cells (**B**). Subtraction of these spectra results in the 1D spectrum of in situ native starch (**C**).

In cells, SS-NMR can also be used to monitor cellular growth under different conditions. Indeed, we have compared various *C. reinhardtii* strains under nitrogen-rich and nitrogen-deprived diets. The effects are such that simple 1D NMR and spectral subtraction are enough to isolate starch signals. For example, after six days of cell culture, the difference spectrum between the *wt* strain and the starchless mutant (*sta 6-1*) leads to an in situ starch spectrum with a fairly good resolution (Figure 7). This type of approach can be used to determine the crystallinity of starch in the storage grains of the microalga in situ, with similar results to purified starch (see Table S1). Similarly, under nitrogen starvation of amylopectin-rich (strain *sta 3-3*) and amylose-rich (strain *st 2-1*) starch producing mutants, starch overproduction is so intense that a subtraction of its spectrum to that of microalgal cells grown in normal medium resulted in an in situ spectrum of starch (data not shown).

Although nitrogen depletion initially leads to detectable starch overproduction, starch signals were comparable to those of normal cells after one month. More than 80% of microalgal cells survived without any addition of carbon or nitrogen sources because starch reserves had become *C. reinhardtii*’s main nutrient.

Living microalgae were introduced in the NMR spectrometer and were monitored by ^13^C SS-NMR under magic-angle spinning. During the course of the experiments, microalgae might die from a combination of spinning, heating and starving, however the structures of most of their constituents remain intact. In cells, ^13^C SS-NMR is thus a versatile approach that enables the identification of starch in microalgae, the comparison of various strains or growth conditions and the study of algal metabolism by monitoring the amount of starch and its progressive degradation throughout the cell life. Starch accumulation, which is a well-known metabolic response to stress, suggests that whole cell SS-NMR experiments could be a useful tool to monitor stress in microalgae.

## 3. Materials and Methods

### 3.1. Materials

^13^C-labelled (99%) sodium bicarbonate was obtained from Cambridge Isotope Laboratories (Tewksbury, MA, USA) or Martek Isotopes LLC (Olney, MD, USA). Natural abundance maize starch, all chemicals used for the growth medium, starch extraction and cell viability assays, such as Percol^®^ and Fluorescein DiAcetate (FDA) were purchased from Sigma-Aldrich (Oakville, ON, Canada).

### 3.2. Strain, Media and Growth Conditions

*Strains*. Wild type strain of *Chlamydomonas reinhardtii 222+* was obtained from the collection of the Institut de Biologie Physico-Chimique (Paris, France). Other strains were obtained from the *Chlamydomonas Resource Center* at the University of Minnesota (http://chlamycollection.org/). Strains *sta 3-3* (CC 2916, soluble starch synthase III mutant [52]), *st 2-1* (CC 2687, granule bound starch synthase mutant [53]) and *sta 6-1* (CC 5373, ADP-glucose phosphorylase mutant [54,55]) are amylose-rich, amylopectin-rich and inhibited starch producing strains, respectively.

*Algal growth*. Tris minimal medium buffered with HCl to pH 7.3 was made as described by Surzycki [56]. Briefly, the medium in polyethylene Erlenmeyer (Nalgene, Thermo Scientific, Waltham, MA, USA) was inoculated with microalgae kept on TAP-medium 1.6% agar plates. The tris-minimal medium was supplemented with 1 g/L of sodium bicarbonate filtered immediately prior to cell culture. For cellular growth, heterotrophic conditions were imposed by purging CO_2_ and O_2_ through gas nitrogen bubbling before sealing the Erlenmeyer. Thereafter, the cells were grown under continuous white light illumination (100 µmol photons·m^−2^·s^−1^) at 23 °C ± 1 °C with gentle agitation (100 rpm). Five days were needed to reach the exponential phase (5.10^6^ cell/mL). Cells were harvested after 1500× *g* centrifugation for 10 min. For ^13^C labelling, labelled sodium bicarbonate (NaH^13^CO_3_) was used as the carbon source at 1 g/L in the Tris minimal medium.

*Whole cell experiments*. For whole cell experiments, microalgal cells were centrifuged, then rinsed twice with a buffer containing 86 mM NaCl and ~50 mg were packed in a 3.2 mm Varian NMR rotor.

*Starch overproduction*. As larger volumes are needed for starch purification, a 300 mL preculture of wild type *C. reinhardtii* was grown to 4 × 10^6^ cells/mL and then 50 mL of this solution was added to 2.5 L of medium contained in a 6 L Erlenmeyer. Again, cells were harvested in the exponential phase (5–6 × 10^6^ cells/mL) after 6 days. Starch overproduction in mutant strains was achieved via nitrogen starvation using the medium described by Ball et al. [22], consisting of the normal growth media simply omitting nitrogen. According to this article, nitrogen depletion is the best compromise, under heterotrophic conditions, between cell production and starch accumulation compared to phosphate and sulphur depletion. This overproduction represents a tenfold final improvement in pure starch yield.

### 3.3. Starch Purification

We adapted the protocols of starch extraction from Buléon and co-workers [11]. Briefly, cells were harvested in the exponential phase by centrifugation for 10 min at 1500× *g* and 4 °C. Cells were rinsed twice with HEPES buffer (HEPES 250 mM, MgCl_2_ 5 mM, sucrose 300 mM, pH 7.3 with EDTA 10 mM, benzamidine 1 mM and PMSF 200 µM as antiproteases) and were then centrifuged again.

The cells pellet was diluted to 10^8^ cells/mL in the same buffer and was disrupted using a homogenizer (EmulsiFlex-C5, Avestin, Ottawa, ON, Canada). Cells were passed through this cell homogenizer 4 times at 10,000 psi and were centrifuged at 2000× *g* for 20 min, thus pelleting big cell debris and starch granules. Starch was washed several times with Milli-q water and was further purified using Percol^®^ (one volume starch for 4 volumes of Percol). The final pellet was considered as pure native starch and was used without further purification for XRD and NMR. For storage that was longer than 3 days, starch was lyophilized. NMR and XRD tests proved that rehydrated starch reaches the same structure than the native one. For NMR experiments, starch was hydrated and ~50 mg was packed in a 3.2 mm Varian NMR rotor.

### 3.4. Starch Retrogradation

A-type starch can be transformed into B-type starch using retrogradation, i.e., recrystallization of the amorphous phase after gelatinization [57]. First, starch was boiled for 15 min in excess water (10% *w*/*w* suspension) at 100 °C in a sealed glass tube. The resulting gel was then slowly cooled and stored at 4 °C. After one month, B-type XRD patterns were observed (Figure S1B), which was in good agreement with the literature [58]. This product was considered as the B-type model starch in this study.

### 3.5. Cell Viability

To test cell viability, fluorescence analysis is performed on a BD Accuri™ C6 flow cytometer (BD Biosciences, Mississauga, ON, Canada). A total of 1000 intact cells were acquired for each sample. This test measures the natural chlorophyll fluorescence and FDA-derived fluorescence generation based on esterase activity level [59]. Briefly, 5 µL of a 1 mM FDA solution was added to 1 mL of cell solution at 2 × 10^6^ cells/mL for 20 min prior to measurement. Thus, cell viability was verified to be above 93% before each whole cell NMR experiment.

### 3.6. Solid-State NMR

All spectra were recorded on a Bruker Avance III-HD (Milton, ON, Canada) operating at a frequency of 150.87 MHz for ^13^C and 599.95 MHz for proton (^1^H) using a Varian 3.2 mm magic-angle spinning (MAS) triple resonance probe (Agilent, Santa Clara, CA, USA). The spinning frequency was set to 15 kHz and the probe was kept at room temperature corresponding to a sample temperature of approximately +35 °C for all experiments. 1D (single-pulse (SP) and cross-polarization (CP)) as well as 2D experiments (Incredible Natural Abundance Double QUAntum Technique (INADEQUATE)) were recorded with nutation frequencies of 60 kHz and 75 kHz for carbon and proton channels, respectively (corresponding to 4.2 and 3.35 µs 90° pulses) [29,30,33,60] and two-pulse phase modulation (TPPM) dipolar decoupling. For CP spectra, 256 scans were typically recorded with a recycling delay of 15 s and an optimized 1 ms contact time (total duration of 1 h). For SP, 128 scans were recorded with 30 s of recycling delay (total duration of 1 h). For 2D experiments, CP or a 2 s long Nuclear Overhauser Effect (NOE) pulse sequence were used to transfer nuclear spin polarization from ^1^H to ^13^C, with a 3 s recycle delay (1 s for NOE), an acquisition time of 25 ms, a total of 768 t1 increments of 32 scans and quadrature acquisition using the states-Time Proportional Phase Incrementation (TPPI) method (total duration of ~20 h). The delay τ during which the J couplings evolve was set to 2 ms. For NOE-INADEQUATE spectra, an NOE delay of 1 s was used. ^13^C chemical shifts were externally calibrated with respect to adamantane fixing the CH_2_ resonance at 38.48 ppm [61]. Spectra were processed using Topspin (Bruker) or Mnova software (Mestrelab Research, Santiago de Compostela, Spain). No line broadening was used on the 1D spectra, while the zero-filling to 1024 points and a sine squared apodization were applied in both dimensions prior to Fourier transform of the INADEQUATE spectra.

### 3.7. X-ray Diffraction

X-ray diffraction was performed on dry or wet samples (~130 mg). Patterns were recorded in transmission mode on a *Bruker D8 Advance* (Milton, ON, Canada) diffractometer operating at 40 mA and 40 V. The Cu K_α_ radiation (*λ* = 1.5406 Å) was selected using a motorized slit of 0.681 nm. Data were recorded in triplicate, with incident angles (2θ) ranging from 2° to 60°, and signals were averaged prior to normalization. Diffractograms were normalized to the same total area under the scattering curve over the Bragg angle range 5–45° (2θ).

### 3.8. Crystallinity Quantification

Crystallinity was assessed by SS-NMR using the method proposed by Lopez and co-workers [62] on 1D CP experiments. Briefly, a positive or negative weighting factor was applied to each resonance for purely crystalline or purely amorphous resonances, respectively. Starch crystallinities are listed in Supplementary Table S1.

## 4. Conclusions

In this work, we presented an unambiguous full assignment of native and retrograded starches in the A and B forms, amylose- and amylopectin-rich starches. The high resolution that was obtained in 2D ^13^C INADEQUATE NMR experiments enabled, for the first time, the assignment of the C_2_, C_3_ and C_5_ sites in different starch samples. This work confirmed differences that were observed previously between various starch types, and also added C_3_ and C_5_ chemical shifts and multiplicity to discriminate between them. Furthermore, full new spin systems were described for amorphous starch and non-reducing end groups of starch. ^13^C SS-NMR spectra also allowed the assessment of starch crystallinity, disorder and dynamics, and could also be used to evaluate the chain length and amylose/amylopectin content.

This study is a step forward in the differentiation between saccharides within a microalgal cell. It is the first one reporting *in-cell* SS-NMR measurements with sufficient resolution to distinguish amorphous, A- and B-type starches, without any time-consuming and potentially altering purification steps. Moreover, we showed that starch crystallinity can be assessed in the cell. The application of this methodology to various *C. reinhardtii* mutants under different growth conditions and the detection of by-products of starch biosynthesis and its metabolism show how this approach could be applicable to the in situ study of other microorganisms. This work also represents a solid base to study physicochemical functionalisation of starch matrices and starch degradation in bioenergy, food industry or drug delivery contexts, for example.

## Figures and Tables

**Table 1:** ^13^C assignments (in ppm) of amylopectin-rich, native retrograded, amylose-rich and native *C. reinhardii* starches ^1^.

	C1		C2		C3		C4		C5		C6
**Amylopectin (A)**	101.8	→	73.6								
	100.6	→	72.5	→	74.2	→	77.9	→	71.9	→	61.6
	99.1	→	71.9								
*Non reducing end group*	100.5	→	72.3	→	73.7	→	70.4	→	73.7	→	61.1
**Retrograded (B)**	100.9	→	72.1	↘				↗	72.4	→	61.0
					74.1	→	77.7				
	100.1	→	72.8	↗				↘	71.5	→	61.6
*Non reducing end group*	100.1	→	72.2	→	73.5	→	70.4	→	73.5	→	61.5
**Amylose (B)**		↗	73.5	↘							
	100.8				74.1	→	77.8	→	71.8	→	61.4
		↘	72.5	↗							
	99.9	→	71.6								
*Non reducing end group*	100.6	→	72.6	→	73.6	→	70.3	→	73.6	→	61.3
*Amorphous*	103.0	→	72.7	→	75.4	→	81.5	→	71.5	→	60.4
***C. reinhardtii wt***	101.5	→	72.1								
**Native starch (A)**	100.5	→	72.6								
	99.3	→	71.9								
					75.0	→	76.9				
*Non reducing end group*	100.4	→	72.4	→	73.6	→	70.2	→	73.6	→	61.4
*Amorphous*	103.0	→	73.0	→	75.3	→	81.7	→	71.5	→	60.2
*By-product 1*	92.7	→	72.4	→	74.9	→	76.9	→	70.4	→	61.4
*By-product 2*	96.7	→	74.9								

^1^ Amylopectin-rich and amylose-rich starches are purified from st 2-1 and sta 3-1 C. reinhardtii strains, respectively. Correlation pathways are determined using NOE-INADEQUATE except for amorphous starch using CP-INADEQUATE. Arrows represent two correlated carbons in the same spin system.

## Supplementary Materials

Supplementary materials can be found online.

## Abbreviations

2D	Two dimensional
SS-NMR	Solid state Nuclear Magnetic Resonance
^13^C	Carbon 13
µm	Micrometer
nm	Nanometer
XRD	X-ray diffraction
1D	One dimensional
g/L	Gram per liter
rpm	Rotation per minute
NaH^13^CO_3_	Sodium bicarbonate
mg	Milligram
mm	Millimeter
mL	Milliliter
mM	Millimole per liter
HEPES	4-(2-hydroxyethyl)-1-piperazineethanesulfonic acid
EDTA	Ethylenediaminetetraacetic acid
PMSF	Phenylmethylsulfonyl fluoride
FDA	Fluorescein DiAcetate
MHz	Mega Hertz
MAS	Magic Angle Spinning
^1^H	Proton
kHz	Kilo Hertz
SP	Single pulse
CP	Cross polarization
INADEQUATE	Incredible Natural Abundance Double QUAntum Technique
µs	Microseconde
TPPM	Two-pulse phase modulation
NOE	Nuclear Overhauser Effect
TPPI	Time Proportional Phase Incrementation
Å	Ångström
DQ	Double quantum
ppm	Parts per million
*wt*	Wild type
*ap*	Amylopectin-rich starch
*as*	Amylose-rich starch
*retro*	Retrograded wild-type starch
*am*	Amorphuous starch
