# Supplementary material for: Unambiguous Ex Situ and in Cell 2D 13C Solid-State NMR Characterization of Starch and Its Constituents
Source: Int J Mol Sci. 2018 Nov 30;19(12):3817. doi: 10.3390/ijms19123817 (PMC6320826; doi:10.3390/ijms19123817)
Supplement: Supplementary file 1 [file ijms-19-03817-s001.pdf]

Supplementary Material

Article

Unambiguous ex situ and *in cell* 2D ^{13}C solid-state NMR characterization of starch and its constituents

Alexandre Poulhazan [1], Alexandre A Arnold [1], Dror E Warschawski [1,2] and Isabelle Marcotte [1,*]

[1] Department of Chemistry, Université du Québec à Montréal, Downtown Station, P.O. Box 8888, Montreal H3C 3P8, Canada; poulhazan.alexandre@courrier.uqam.ca; arnold.alexandre@uqam.ca; marotte.isabelle@uqam.ca.

[2] Laboratoire de Biologie Physico-Chimique des Protéines Membranaires, UMR 7099, CNRS, Université Paris Diderot and IBPC, 13 rue Pierre et Marie-Curie, 75005 Paris, France; Dror.Warschawski@ibpc.fr.

* Correspondence: marotte.isabelle@uqam.ca; Tel.: +1514 987 3000 # 5015

Received: date; Accepted: date; Published: date

Table of content

Table S1. Crystallinity evaluation using the method suggested by Lopez *et al*. (2008) [1] on 1D CP NMR experiments for extracted and *in situ* starch from wild-type (*wt*), amylopectin-rich starch (*ap*), amylose-rich starch (*as*), native retrograded (*retro*) and dry amorphous amylose (*am*).

Figure S1. XRD diffractograms of dry (left column) and hydrated (right column) samples. XRD diffractograms are acquired on amylopectin-rich (A-type) (**A**), native retrograded (B-type) (**B**), amylose-rich (**C**), native *C. reinhardtii* (**D**) and amorphous (**E**) starches.

Figure S2. Overlapped CP-INADEQUATE of pure *C. reinhardtii* native starch (**black**) and amorphous dry amylose (**red**). Dashed lines represent spin system of amorphous starch determined using CP-INADEQUATE on native *C. reinhardtii* starch. Here CP is used instead of NOE-INADEQUATE because the polarization transfer lead to a better resolution in the amorphous region.

Figure S3. 1D cross-polarisation ^{13}C solid-state NMR spectra of amylopectin (**A**) and amylose (**B**) starches from *C. reinhardtii* strains *st 2-1* and *sta 3-3*, respectively.

Figure S4. Overlapped NOE-INADEQUATE of pure *C. reinhardtii* amylopectin-rich starch (**black**) and amylose-rich starch (**red**). Dashed lines represent spin system of B-type starch while continuous lines are A-type starch. Here, amylose starch is not crystalline enough to make a clear difference with the highly crystalline amylopectin-rich starch.

Figure S5. Overlapped NOE-INADEQUATE of pure *C. reinhardtii* native (**red**) and amylopectin-rich (**black**) starches.

Table S1. Crystallinity evaluation using the method suggested by Lopez and co-workers[1] on 1D CP ^{13}C NMR experiments for extracted and *in situ* starch from wild-type (*wt*), amylopectin-rich starch (*ap*), amylose-rich starch (*as*), native retrograded (*retro*) and dry amorphous amylose (*am*).

		wt	ap	as	retro	am
NMR	*extracted*	62.9	71.2	38.0	54.5	0.1
	in situ	62.3	69.9	40.0		

[1] Lopez-Rubio, A.; Flanagan, B. M.; Gilbert, E. P.; Gidley, M. J. A novel approach for calculating starch crystallinity and its correlation with double helix content: a combined XRD and NMR study. *Biopolymers* **2008**, *89*, (9), 761-8.

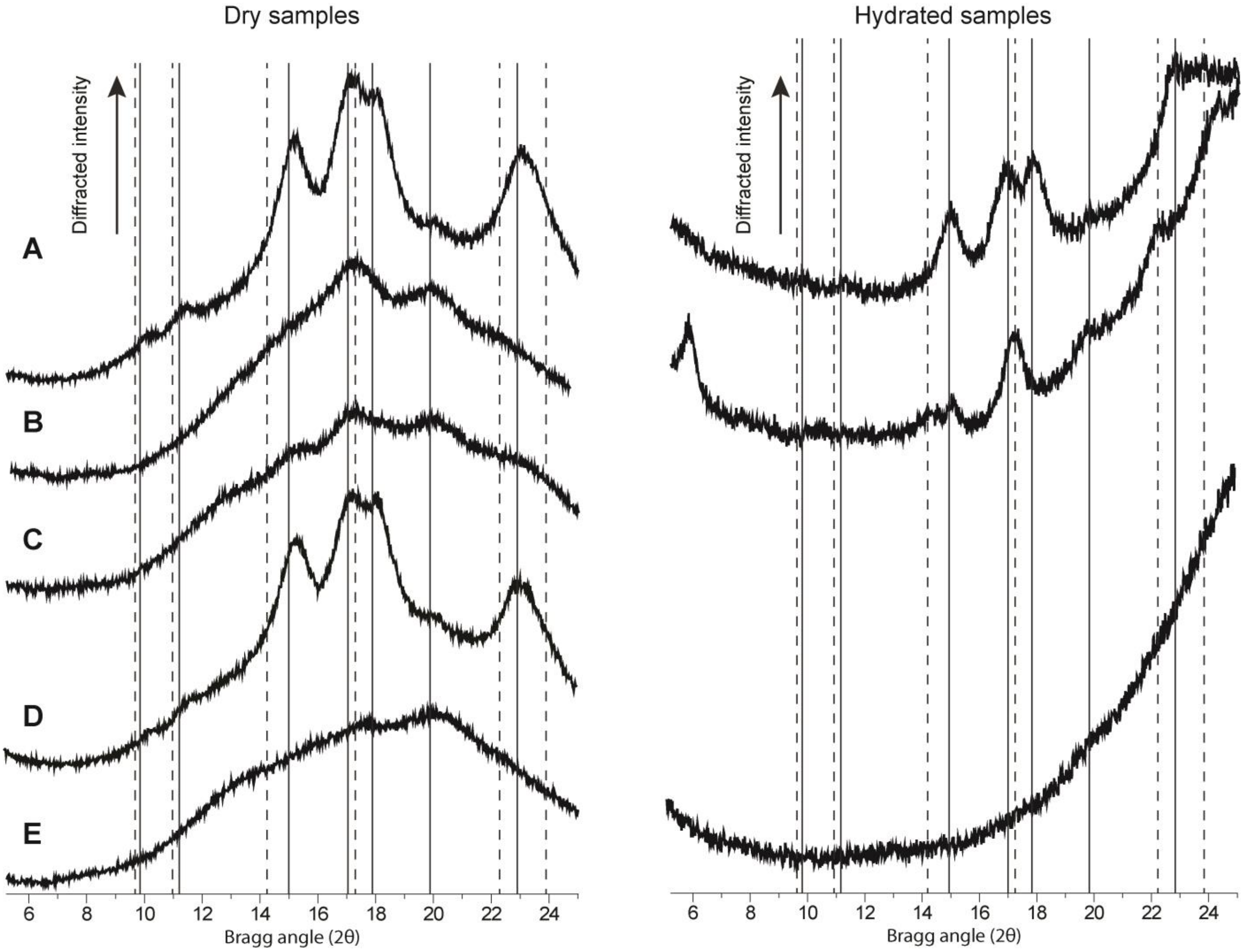

Figure S1. XRD diffractograms of dry (left column) and hydrated (right column) samples. XRD diffractograms are acquired on amylopectin-rich (A-type) (**A**), native retrograded (B-type) (**B**), amylose-rich (**C**), native *C. reinhardtii* (**D**) and amorphous (**E**) starches.

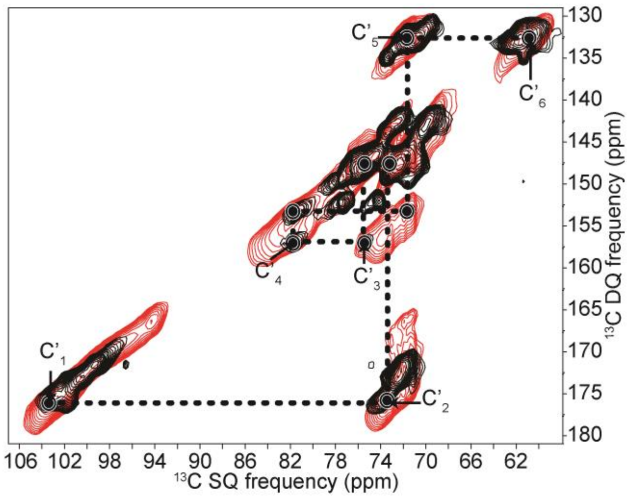

Figure S2. Overlapped CP ^{13}C INADEQUATE spectra of pure *C. reinhardtii* native starch (**black**) and amorphous dry amylose (**red**). Dashed lines represent spin system of amorphous starch determined using CP-INADEQUATE on native *C. reinhardtii* starch. Here CP is used instead of NOE-INADEQUATE because the polarization transfer leads to a better resolution in the amorphous region.

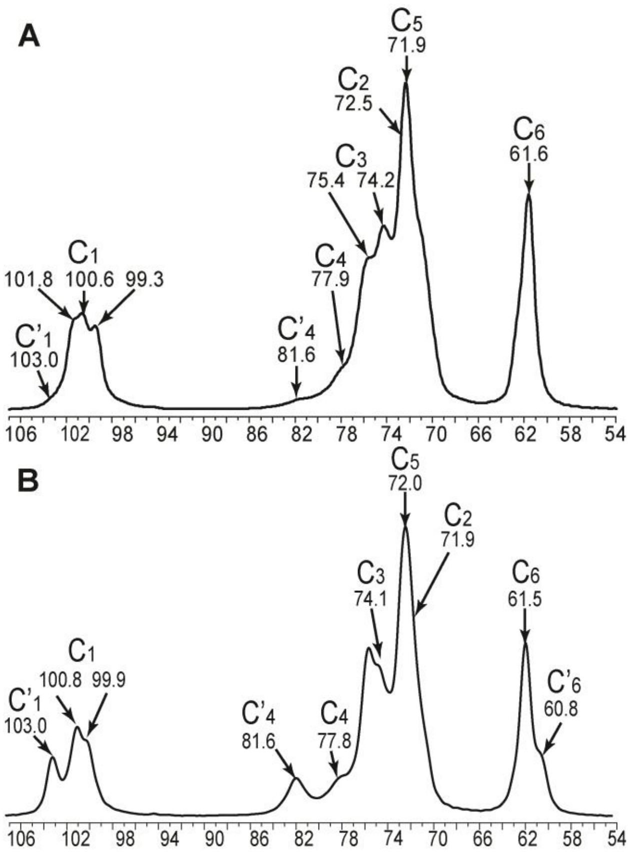

Figure S3. 1D CP ^{13}C solid-state NMR spectra of amylopectin (**A**) and amylose (**B**) starches from *C. reinhardtii* strains *st 2-1* and *sta 3-3*, respectively.

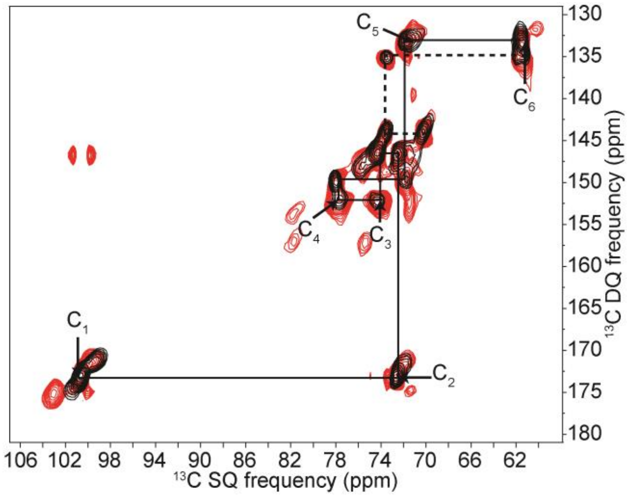

Figure S4. Overlapped ^{13}C NOE-INADEQUATE of pure *C. reinhardtii* amylopectin-rich starch (**black**) and amylose-rich starch (**red**). Dashed lines represent spin system of B-type starch while continuous lines are A-type starch. Here, amylose starch is not crystalline enough to make a clear difference with the highly crystalline amylopectin-rich starch.

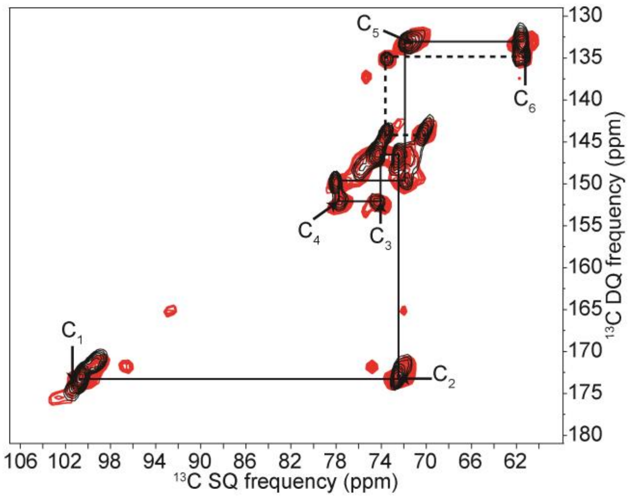

Figure S5. Overlapped ^{13}C NOE-INADEQUATE of pure *C. reinhardtii* native (**red**) and amylopectin-rich (**black**) starches.
